# Umbelliprenin from *Ferula szowitsiana* Activates both Intrinsic and Extrinsic Pathways of Apoptosis in Jurkat T-CLL cell line

**Published:** 2013

**Authors:** Omid Gholami, Mahmood Jeddi-Tehrani, Mehrdad Iranshahi, Amir Hassan Zarnani, Seyed Ali Ziai

**Affiliations:** a Physiology and Pharmacology Department, School of Medicine, Sabzevar University of Medical Sciences, Sabzevar, Iran.; b Monoclonal Antibody Research Center, Avicenna Research Institute, ACECR, Tehran, Iran.; c Biotechnology Research Center and School of Pharmacy, Mashhad University of Medical Sciences, Mashhad, Iran.; d Nanobiotechnology Research Center, Avicenna Research Institute, ACECR, Tehran, Iran.; e Pharmacology Department, Faculty of Medicine, Shahid Beheshti University of Medical Sciences, Tehran, Iran.*. *

**Keywords:** Umbellprenin, CLL, Apoptosis, Caspase, Western blot

## Abstract

Umbelliprenin is a prenylated compound, which belongs to the class of sesquiterpene coumarins. It is extracted from dried roots of *Ferula szwitsiana* collected from the mountains of Golestan forest (Golestan Province, north of Iran). Induction of apoptosis in Jurkat T-CLL cells has been previously shown. In this study, effect of umbelliprenin on proapoptotic caspases (caspase-8 and -9) and antiapoptotic Bcl-2 family protein was studied. Jurkat cells were incubated with umbelliprenin. Cells were then lysed and activation of proteins was studied by Western blot analysis.

In this study, we showed that umbelliprenin activates intrinsic and extrinsic pathways of apoptosis by the activation of caspase-8 and -9 respectively. Inhibition of Bcl-2 was also shown. In conclusion, umbelliprenin induced apoptosis in Jurkat cells through caspase-dependent apoptosis pathway.

## Introduction

Chronic lymphocytic leukemia (CLL) is one of the most prevalent types of leukemia in western countries. CLL, a monoclonal B-cell malignancy with a low level of proliferation, is characterized by a progressive accumulation of mature-appearing but functionally incompetent, malignant B lymphocytes. 

**Figure 1:**
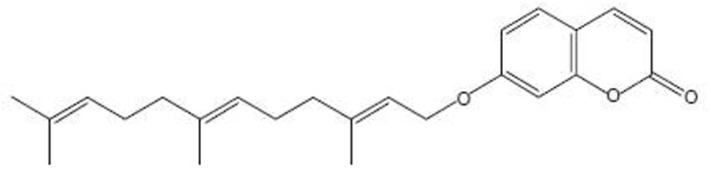
Chemical structure of umbelliprenin

Umbelliprenin (Figure 1) is a naturally occurring prenylated coumarin, which is synthesized by various *Ferula* species. 

Umbelliprenin has been found in various plant species consumed as food or used for food preparation, such as celery, *Coriandrum sativum*,* Citrus limon *and* Angelica archangelica*. Iranshahi* et al.* reported that umbelliprenin inhibits red pigment production in *Serratia marcescens* (2) and Cravotto* et al.* found that this coumarin can inhibit squalene-hopene cyclase (an enzyme taking part in sterol synthesis) (3). Reduction of matrix metalloproteinase activity (4), and antileishmanial activity against promastigotes (5) by umbelliprenin have also been shown. Induction of apoptosis in human M4Beu metastatic pigmented melanoma cells by umbelliprenin have been reported by Barthomeuf *et al.* (6).

Induction of apoptosis by umbelliprenin in Jurkat T-CLL cells has already been shown. We previously incubated Jurkat T-CLL and Raji B-CLL cells with various concentrations of umbelliprenin at different times, and umbelliprenin induced apoptosis in both cell lines in a dose- and time- dependent manner. Moreover, CLL cells were more susceptible to apoptotic effect of umbelliprenin than normal Peripheral Blood Mononuclear Cells (PBMCs) (7). However, the mechanism of apoptosis remains to be studied. 

Given the ability of umbelliprenin to induce apoptosis in Jurkat cells, in this study we investigated the mechanism of apoptosis induction in Jurkat cells by umbelliprenin. 

## Experimental

*Plant material and umbelliprenin isolation*

Umbelliprenin (C_24_H_30_O_3_, MW: 366) was purified (> 95%) as previously described (5) from dried roots of *F. szowitsiana* D.C collected from the mountains of Golestan forest. A voucher specimen of the roots (No. M1001) was deposited at the Department of Pharmacognosy and Biotechnology, Faculty of Pharmacy, Mashhad University of Medical Sciences. Umbelliprenin was diluted in DMSO. Immediately before use, it was diluted in the culture medium to obtain a final DMSO concentration of 0.5% (v/v).

*Cell culture*

Jurkat cells were prepared from National Cell Bank of Iran (Pasteur institute, Tehran, Iran). Cells were grown in RPMI 1640 culture medium containing 10% fetal bovine serum (FBS), penicillin (10,000 U/mL) and streptomycin (10 mg/mL) in several culture flasks in a CO_2_ (5%) incubator at 37 °C and 95% humidity, until totally 50×10^6^ cells. Cells were then frozen in FBS containing 10% dimethyl sulfoxide (DMSO) and stored in liquid nitrogen (5×10^6^ cells/vial). The viability of cryopreserved cells was determined by trypan blue staining immediately upon thawing. Only cells whose viability exceeded 93% (range, 93.4%-99%) were used in this study.

*Western blot analysis*

Jurkat cells were incubated by umbelliprenin (50 µM) in 37 ºC and 5% CO_2_ for 3, 6 and 16 h. After that cells were collected and lysed with the lysis buffer (EDTA 0.5 M 1 mL, Tris–HCl pH 7.4 50 mL, NaCl 0.88 g, NaF 0.0042 g, Na_4 _P_2 _O_7 _0.89 g, SDS 0.1 g, Triton 1 mL, glycerol 1 mL, protease inhibitor cocktail I (1X; Roche), phosphatase inhibitor cocktail II (1X; Sigma)). Protein concentration was determined using the Bradford method (8). Cell lysates containing 20 µg of total protein were loaded onto 12% SDS–polyacrylamide gels with Tris/glycine running buffer and transferred to polyvinylidene difluoride (PVDF) membranes (Roche USA). Each membrane was blocked with blocking buffer (5% skim milk, NaCl 8.7 g, Tris–Base 6.05 g and D.D.W. to 1000 mL pH 7.4) for 1 h at room temperature and incubated with the primary antibody (anti-caspase-3 Rabbit mAb. 1:1000, anti-caspase-8 Rabbit mAb. 1:1000, anti-caspase-9 Rabbit Ab. 1:1000, anti-Bax Rabbit Ab. 1:1000 and anti-Bcl-2 Rabbit Ab. 1:1000 (Cell Signaling), diluted in 5% skim milk) at 4 °C overnight. After washing with Tris–buffered saline containing 0.1% Tween-20, the membrane was incubated with an Anti-rabbit IgG antibody conjugated with horseradish peroxidase (1:3000, (Cell Signaling), diluted in 5% skim milk, NaCl 8.7 g, Tris–Base 6.05 g and D.D.W. to 1000 mL pH 7.4) at room temperature for 1 h. The blots were incubated with antibodies that recognize *β*-actin (mouse mAb., Avicenna Research Institute, Tehran, Iran) as loading control. The signal was detected using an enhanced chemiluminescence Western blotting detection system (Amersham Bioscience). 

*Flowcytometry analysis of cleaved caspase 8*

Jurkat cells were incubated by umbelliprenin (50 µM) in 37 ºC and 5% CO_2_ for 3, 6 and 16 h. After that cells were collected and washed with phosphate-buffered saline (PBS 1X). Fixative buffer (0.01% formaldehyde in PBS) was added to cells and stored on ice for 10 min. After addition of PBS- tween 0.2% to cells, they were incubated in room temperature for 15 min. Cells were incubated with primary antibody (anti-cleaved caspase-8 rabbit mAb. 1:100 (Cell Signaling) diluted in PBS-tween) for 1 h on ice. After washing with PBS-tween, secondary Ab. (Sheep anti-rabbit-FITC 1:50, Avicenna research institute, Tehran, Iran) was added and incubated for 1 h. Changes in cleaved caspase-8 protein level was detected by flow cytometry method (Partec Flomax) using FL-1 channels.

*Statistical analysis*

One way ANOVA test was used for statistical analysis. The p-value was considered significant when it was less than 0.05.

## Results

*Umbelliprenin induces caspase-dependent apoptosis of Jurkat cells*

**Figure 2:**
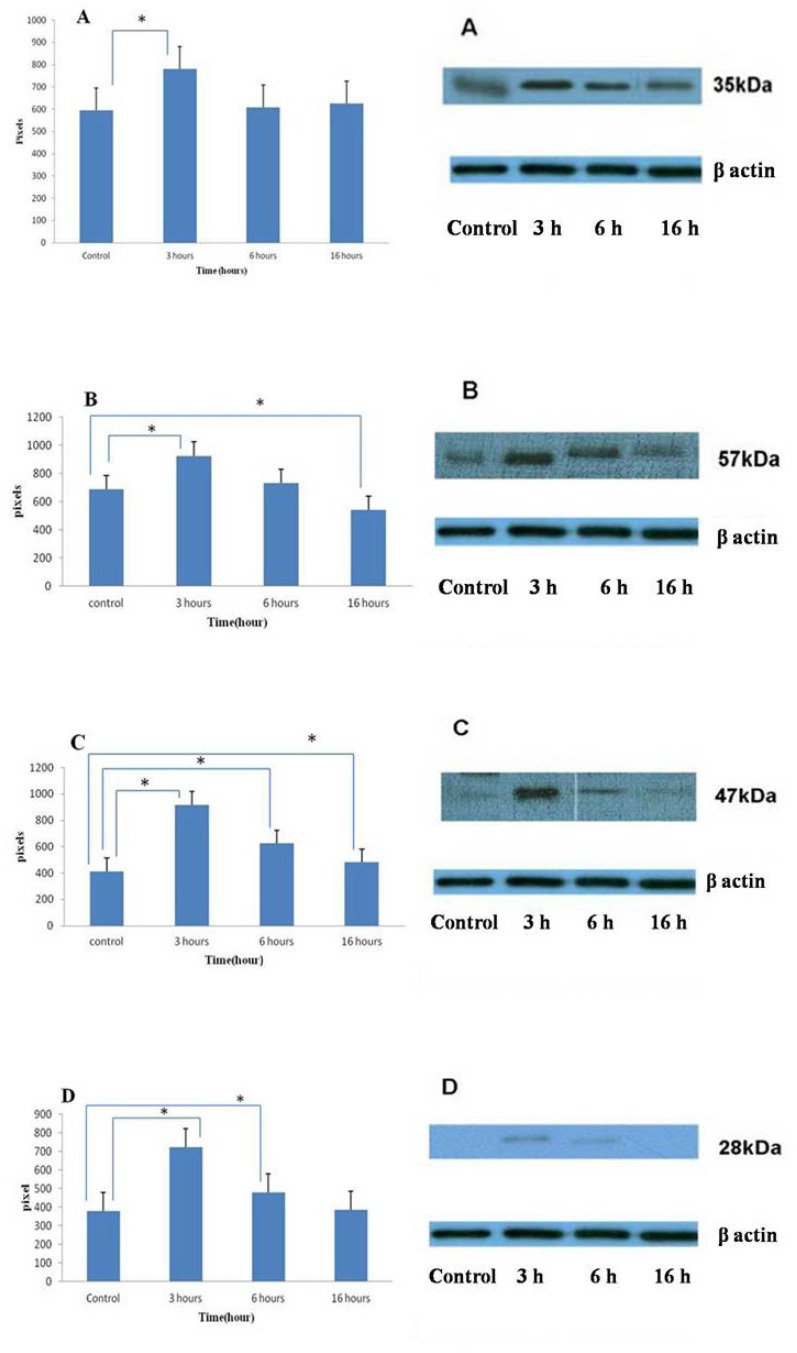
Changing in protein expression by umbelliprenin (50 μM) on Jurkat cells after 3, 6, 16 h of incubation. Umbelliprenin activates the production of procaspase-3 (A), -8 (B), -9 (C) and Bcl-2 (D) at 3 h and decreases them after that time. *β*-actin was used as a loading control. The ratio of each protein to *β*-actin was calculated each time and showed as column chart. Data are shown as mean ± standard deviation. *p < 0.05 in compared columns are based on Tukey tests in ANOVA analysis

Caspase-3 is an effector caspase that plays a central role in the mitochondrial-mediated cell death pathway and is responsible for the breakdown of several cellular components involved in DNA repair and regulation. To determine whether apoptosis induced by umbelliprenin was associated with activation of caspase-3, cells were incubated with umbelliprenin at different times, and then analyzed by Western blotting method. Results showed that umbelliprenin first induced significant increase in procaspase-3 amounts after 3 h of treatment, after which procaspase-3 was activated to caspase-3; thus procaspase-3 amounts were decreased from 3 h to 16 h of treatment (Figure 2a).

To further characterize umbelliprenin-induced apoptosis, we examined whether umbelliprenin activates the extrinsic or intrinsic apoptotic pathway in CLL cells. To determine which apoptotic pathway is activated by umbelliprenin, we examined patterns of proteolytic processing of caspase-8 and -9, the apical proteases in the extrinsic and intrinsic pathways, by Western blot analysis, respectively. Levels of procaspase-8 increased after 3 h of treatment, which meant that umbelliprenin first induced procaspase-8, after which procaspase-8 was activated to caspase-8 from 3 h to 16 h of treatment so the band density decreased significantly (Figure 2b). Levels of procaspase-9 also increased upon umbelliprenin treatment after 3 h. However, from 3 h to 16 h treatment levels of procaspase-9 decreased. These data show that procaspase-9 was activated to caspase-9 from 3 h to 16 h of treatment by umbelliprenin (Figure 2c). These data also suggest that both caspase-8 and -9 were activated by umbelliprenin. Thus, umbelliprenin activated intrinsic and extrinsic pathways of apoptosis.

To further examine whether important proapoptotic and antiapoptotic regulatory proteins could be modulated by umbelliprenin in Jurkat cells, we analyzed Bcl-2 and Bax levels by Western blotting. Bcl-2 is an antiapoptotic regulatory protein that is overexpressed in B-CLL cells (11, 12). Western blot analysis revealed that exposure of CLL cells to umbelliprenin first increased Bcl-2 levels after 3 h of treatment. But from 3 h to 16 h treatment, levels of Bcl-2 decreased (Figure 2d). In contrast, the expression of Bax, a protein that can promote apoptosis, could not been detected by Western blot analysis following umbelliprenin treatment. The combination of caspase-3, caspase-8 and caspase-9 activation, and down-regulation of Bcl-2 explains, in part, the onset of apoptosis induced by umbelliprenin in Jurkat cells.

*Levels of cleaved caspase-8 increased after umbelliprenin treatment*

**Figure 3:**
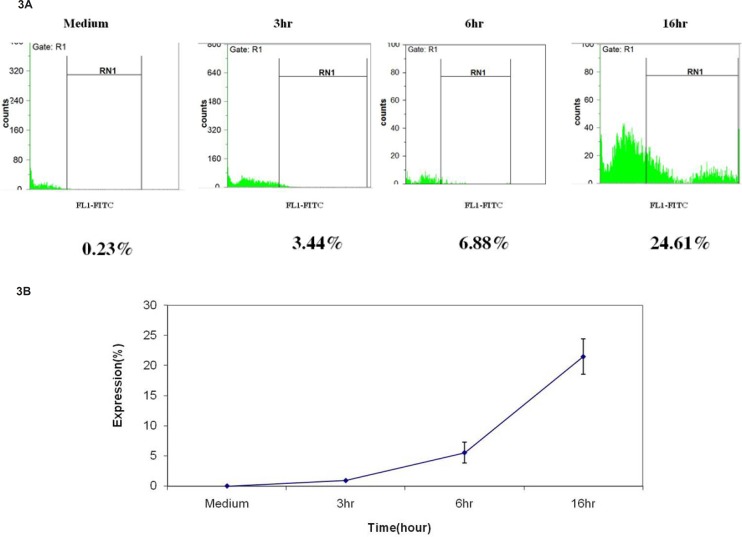
Increase in cleaved caspase-8 protein expression after incubation of Jurkat cells with umbelliprenin (50 μM) for 3, 6 and 16 h. Data are shown as mean ± standard deviation

To further characterize the cytotoxic action of umbelliprenin, we examined the up-regulation of cleaved caspase-8 by flow cytometry. For this, Jurkat cells were incubated with umbelliprenin at different times, and then analyzed by flow cyotmetry using FITC chemiluminescence. Results showed that umbelliprenin increases cleaved caspase-8 levels significantly (Figure 3).

## Discussion

Our study investigated the mechanism of apoptosis induction in Jurkat cells by umbelliprenin, a terpenyloxy coumarin from *F. szowitsiana*. Other herbal medicines that have been extracted from genus *Ferula* show cytotoxic activity against cancerous cells. Elaeochytrin A from *F. elaeochytris* has shown cytotoxic activity in K562R (imatinib-resistant) human chronic myeloid leukaemia and DA1-3b/M2BCR-ABL (dasatinib-resistant) mouse leukemia cell line (9).

 Xanthoangelol, a major chalcone constituent of the stem exudates of *Angelica keiskei* (Umbelliferae), induces apoptotic cell death by activation of caspase-3 in Jurkat cells through a mechanism that does not involve Bax/Bcl-2 signal transduction (10). Imperatorin, a furanocoumarin from the roots of *Angelica dahurica *(Umbelliferae), induces apoptosis in human promyelocytic leukaemia, HL-60 cells. Further studies showed that the cytochrome c/caspase-9 pathway was responsible for imperatorin-induced apoptosis. *Pae et al.* have reported that imperatorin can depolarize mitochondrial membrane, down-regulate Bcl-2, release cytochrome c from mitochondria, and activate caspase-9 and caspase-3 (11). 

 The death circuitry in mammalian cells has two major apoptotic pathways (12-13). One is a receptor mediated pathway that activates caspase-8 (extrinsic pathway), whereas the other involves casapse-9 (intrinsic pathway) (12-13). Here, we have demonstrated that umbelliprenin induces the activation of caspase-9. We have also demonstrated that umbelliprenin-induced apoptosis come along with caspase-8 activation. These results suggest that umbelliprenin- induced apoptosis may be linked to mitochondrial function and receptor-mediated reaction.

The Bcl-2 family consists of anti-apoptotic (like Bcl-2) and proapoptotic (like Bax) proteins that interact with each other to regulate the survival or death of the cells (14). Bcl-2 is found in the membranes of mitochondria, the endoplasmic reticulum and the nucleus (15). Bcl-2 has been linked to the homeostasis of mitochondrial membranes and has been shown to block the release of cytochrome c and inhibit the activation of caspases (14-15). We analyzed whether this substance could affect Bcl-2 and Bax expression. We found that Bcl-2 levels were increased after treatment with umbelliprenin in Jurkat cells at 3 h but reduced after that time. Bax levels were measured by Western blotting analysis but no results were detected. These results suggest that umbelliprenin treatment increased both proapoptosis (caspases 3, 8 and 9) and antiapoptosis (Bcl-2) pathways and the net effect was 36% apoptosis in these cells as shown before (7).

We demonstrated that umbelliprenin induces apoptosis in Jurkat cells by caspase-mediated programmed cell death. Umbelliprenin activates intrinsic and extrinsic pathways of apoptosis by activation of caspase 9 and caspase 8 proteins respectively. 
